# Infection process, viability and survival of cotton rust (*Phakopsora gossypii*) under different storage conditions

**DOI:** 10.7717/peerj.21412

**Published:** 2026-07-14

**Authors:** Valarmathi Pandian

**Affiliations:** Department of Plant Pathology, ICAR-Central Institute for Cotton Research (CICR), Regional Station, Coimbatore, Tamil Nadu, India

**Keywords:** Scanning electron microscopy (SEM), *Phakopsora gossypii*, Host range, Urediniospores, Appressoria

## Abstract

Cotton rust, caused by the biotrophic fungus *Phakopsora gossypii*, is an emerging disease in cotton that has led to significant yield losses in cotton cultivated areas of India. Considering the current significance of rust and the need for additional basic information about its causal agent for better disease control, this study aimed to determine the infection process of the pathogen in cotton leaves using Scanning Electron Microscopy (SEM). By 42 hours after inoculation (HAI), the urediniospores of rust pathogen had germinated by producing a germ tube and an appressorium capable of directly penetrating the leaf cuticle. By 20 days after inoculation (DAI), closed uredia with urediniospores appeared on the abaxial leaf surface. These uredia start to open 25 DAI and produced numerous urediniospores at 35 DAI. The results of this study provide new insights into the infection process of *P. gossypii* in cotton leaves, potentially aiding in the development of effective strategies for controlling rust disease. Urediniospore germination began early (25.30% at 4 hours) and increased over time, reaching 69.40% after 8 hours. Maximum germination (94.35%) occurred at 72 hours. At 25 °C, maximum germination (96.40%) was recorded after 24 hours. Higher temperature showed reduced germination: 36.12% at 30 °C, 13.54% at 35 °C, and 2.15% at 40 °C, indicating that temperature above 25 °C are unfavorable for urediniospore germination.

## Introduction

Cotton, India’s leading fibre crop, plays a crucial role in both its agricultural and industrial economy. Commercial cultivation of the versatile crop is practiced in the temperate and tropical regions of more than 70 countries worldwide. In India, cotton makes up to 14%–16% of India’s overall agricultural crop production. Throughout the growing period, cotton crop encounters a wide range of foliar related diseases like leaf spots, blights, grey mildew and rust. In India, apart from these foliar diseases, boll rot caused by fungal and/ or bacterial pathogens, diseases caused by bacterial and viral pathogens, have been documented which led to yield reductions ranging from 20 to 30% (Monga, Lakshmi & Prakash, 2013).

However, an ongoing challenge has arisen with the outbreak of a cotton rust epidemic incited by *Phakopsora gossypii* (Arth.) Hirat. In some cases, it has been reported to reduce cotton yields by as much as 24%, as observed in Coimbatore (Johnston, 1963). This disease has caused significant losses, reducing the total yield by 30 to 40% in most Bt cotton hybrids cultivated during the period of 2009–2010 (Pindikur et al., 2012). Estimated avoidable yield losses from rust were 21.7% in Bunny Bt (Monga, Lakshmi & Prakash, 2013) and 34.05% in RCH 2 BG II (Bhattiprolu, 2015). Cotton rust occurs widely throughout tropical and subtropical regions worldwide and can lead to substantial yield reductions under favourable weather conditions. The disease usually develops during the dry months from December to March and has been recorded in states like Karnataka, Andhra Pradesh, and Gujarat (Puri et al., 1998). At present, control of cotton rust mainly depends on fungicide use, which raises production costs for farmers who prefer cultivating resistant cotton varieties. This situation poses a major challenge for the cotton industry and highlights the need for more sustainable approaches to manage rust effectively (Kirkpatrick & Rothrock, 2001; Pindikur et al., 2012). *Phakopsora gossypii* has emerged as an important constraint to cotton production outside India, particularly in Brazil and other tropical regions, where it can cause significant yield and fiber quality losses under warm and humid conditions (Araujo et al., 2015). *Phakopsora pachyrhizi*, the causal agent of soybean rust, is one of the most destructive foliar pathogens of soybean worldwide, capable of causing severe yield losses under favorable environmental conditions (Hartman, Miles & Frederick, 2015; Godoy et al., 2016). Emergence of the plant pathogenic fungus *Phakopsora euvitis* in Brazil and Australia, was identified as the causal agent of Asian grapevine leaf rust (Primiano et al., 2017).

Rust fungi have a complex life cycle with specialized spores. In dikaryotic rusts, sexual reproduction produces teliospores, leading to karyogamy, meiosis, and four haploid basidiospores. The basidiospores then infect either the original host plant or, in the case of heteroecious rusts, a different host species. Only the uredinial stage of *P. gossypii* has been observed, therefore this rust is believed to be autoecious, and despite searching the telial stage has not been observed (Kakishima, Hosaka & Hosoya, 2023). For *Phakopsora pachyrhizi*, information on sexual reproduction is limited. While teliospores have been observed in Asia on various hosts, including soybeans, their germination has never been documented in nature (Bromfield & Hartwig, 1980). Rust fungi are obligate biotrophic pathogens that require living host tissue to grow and reproduce. They survive either within their living hosts or for a period outside their host as spores that must infect their hosts again. To persist into the subsequent growing season, many rust pathogens of crop plants require a living host that serves as a green bridge (Staples, 2003). It was observed that the *Vigna mungo* which exhibited the highest susceptibility to *Phakopsora pachyrhizi*, displaying the maximum average number of lesions per square centimetres of leaf area and the most intense sporulation (do Vale, Chaves & Zambollm, 1986). Hegde (2001) documented the infectivity of *P. pachyrhizi* on various crops, including pulse crops, oilseeds, bhendi and sugarcane. Notably, *Phaseolus vulgaris and Psophocarpus tetragonolobus* were identified as particularly susceptible, showing the maximum quantity of pustules per leaf. Wherein, *Macrotyloma uniflorum, Saccharum officinarum, Cassia*, *Cyperus rotundus* and *Stylonthus* exhibited the lowest average number of pustules per leaf. Cotton leaves developed characteristic rust pustules of *P. gossypii*, indicating that the uredospores were viable and infective under the prevailing conditions. Among the thirteen non-cotton host species evaluated, nine exhibited infection, with pustules containing uredospores of *P. gossypii*. The susceptible hosts included *Arachis hypogaea, Abelmoschus esculentus, Vigna radiata, Vigna unguiculata, Phaseolus vulgaris, Glycine max, Helianthus annuus*, and *Macrotyloma uniflorum*, all of which developed typical rust symptoms. In contrast, *Sesamum indicum, Cajanus cajan, Lablab purpureus Cyamopsis tetragonoloba* and *Cicer arietinum* did not exhibit any pustule formation (Pindikur et al., 2012).

Germination involves the production of a germ tube and an appressorium, a process influenced by environmental conditions. Germination remarkably decreases when spores are exposed to 28.5 to 42.5 °C. Urediniospore viability, the ability to germinate and infect, is also influenced by environmental conditions during the intermittent time between release from the uredinium and coming into contact with susceptible host tissue. Mallaiah & Rao (1979) reported that *Puccinia arachidis* urediniospores germinate within 2 h with maximum germination occurred within 6 h. Pindikur et al. (2012) reported that the highest germination rate (95.62%) of *P. gossypii* urediniospores was observed after 24 h of storage at 25 °C. Further it was observed that with the higher temperature (30 °C), it resulted with lower germination of spores (35.10%) with incubation period of 24 h. Soybean leaf extract agar supported the highest urediniospore germination and germ tube elongation. Maximum *P. pachyrhizi* urediniospore germination occurred at temperatures of 21.8–22.3 °C, while optimal germ tube growth was observed at 21.4–22.1 °C. Peak urediniospore germination was recorded after 6.4 h of exposure, whereas maximum germ tube length was attained after 7.7 h (Blum et al., 2015).

Temperature plays a crucial role in influencing the viability of rust spores. Temperature is the key environmental factor affecting spore germination-other factors like humidity and light are also important. *P. pachyrhizi* urediniospores germinate between 8 to 33 °C. Furthermore, urediniospore germination reduced to 12.89% at 35 °C and 1.13% at 40 °C, suggesting temperatures above 25 °C are unfavourable for its growth. Temperature and relative humidity strongly influenced urediniospore survival, as reflected by their germination capacity. Viable *P. pachyrhizi* urediniospores could be recovered from infected soybean leaves stored at room temperature (23–24 °C and 55–60% RH) for up to 18 days. In contrast, freshly collected urediniospores that were desiccated for 12 h prior to storage and then maintained at room temperature remained viable for as long as 30 days. In relative humidity studies, urediniospores obtained from inoculated leaf tissues maintained at 85% RH [for up to 30 days] exhibited the highest germination rates when compared with both higher and lower humidity levels (Twizeyimana & Hartman, 2010).

Environmental conditions, including temperature and humidity, influence not only urediniospore germination and survival but all processes in the disease development cycle including infection, latency and symptom development. Rust disease development is inhibited below 14 °C and stops entirely below 9 °C (Tschanz, Wang & Tsai, 1986). Additionally, disease development requires relative humidity above 80% for a period of 4–6 h, and under dry leaf conditions the infectivity of the spores declines after approximately 8 days (Melching et al., 1989). Urediniospore viability, the ability to germinate and infect, is also influenced by environmental conditions during the intermittent time between release from the uredinium and coming into contact with susceptible host tissue. Munde & Mayee (1979) reported that there is an increase in incubation period with increase in temperature. Their viability reduces within 5 days at 4 to 5 °C but can last up to 27 days at 9 °C or higher. Further, Benagi (1991) described that maximum germination of *Puccinia arachidis* urediniospores occurs with the requirement of incubation period of 6 h. The cotton rust disease *P. gossypii* occurred under field conditions at 31.5 °C maximum temperature, 22.0 °C minimum temperature, 87% RH I, 50% RH II, 4.5 SSH, 5.9 km/h wind speed and reached maximum intensity at 30.0 °C maximum temperature, 21.1 °C minimum temperature, 84% RH I, 52.0% RH II, 4.7 SSH, 5.7 km/h wind speed in Bt hybrids (RCH659 BG II and Suraj Bt) (Valarmathi, Kanjana & Sankaranarayanan, 2025).

According to Vittal et al. (2014), development of urediniospore, initiation of appressorium and penetration of rust pathogen on soybean leaves occur by 24 h after inoculation. Appressoria enable many fungi to penetrate the cuticle of the host to further initiate the process of infection. While some fungi, which causing powdery mildews and anthracnose, use cutinases for direct penetration, most rely on the strong osmotic pressure of appressoria (Dean, 1997; Ludwig et al., 2014; Mendgen & Deising, 1993). Urediniospores of many rust fungi germinate and penetrate indirectly *via* stomata (Dean, 1997; Mendgen & Deising, 1993). Edwards & Bonde (2011) noted that *P. pachyrhizi* uses mechanical force and digestive enzymes for penetration. Chang, Miller & Hartman (2014) and Loehrer et al. (2014) found that turgor pressure in *P. pachyrhizi* appressoria is independent of melanin biosynthesis. Araujo et al. (2015) observed that pre-penetration events of *P. gossypii* on cotton plants are similar to those of soybean rust pathogen. In addition to *P. pachyrhizi* and *P. gossypii*, *Austropuccinia psidii* develops an appressorium at the tip of a short germ tube, from which an infection peg penetrates between epidermal cells, leading to the establishment of an intercellular mycelium with haustoria within the host leaf tissue (Hunt, 1968).

Recognizing the importance of rust diseases in the context of ongoing challenges in cotton and the need for deeper fundamental understanding to enable the development of effective control strategies, this study investigated the infection mechanism of *Phakopsora gossypii* using Scanning Electron Microscopy (SEM), along with recent field surveys for disease severity and intensity, evaluations of urediniospore survival, viability and its host range.

## Materials and Methods

A survey was carried in the cotton growing areas of Coimbatore to know the occurrence and spread of rust disease during the cropping season of 2021–2023. In the surveyed cotton fields (for each variety/hybrid examined), twenty-five plants were selected at random and the intensity/severity of the disease was recorded at the period of 120 to 140 days after seeding using a 0–4 scale as given by Raj (1988) as given here below.

**Table:** 

**Numerical rating**	**Leaf area covered (%)**
0	No visible symptoms
1	Few small pustules covering up to 10% of leaf area
2	Moderate number of pustules covering 11–25% of leaf area
3	Numerous pustules covering 26–50% of leaf area
4	Severe infection with pustules covering more than 50% of leaf area and associated leaf damage

Percent disease index (PDI) was calculated (Wheeler, 1969) by using this formula:

Percent disease index = Sum of numerical ratings/number of leaves observed × 100/maximum disease rating.

A host range study was conducted to determine the pathogen’s ability to infect plant species other than cotton and to evaluate its survival on non-cotton hosts. This study tested various host species’ responses to the pathogen under greenhouse conditions. Five plants with ten leaves in each plant (35 days old) were inoculated using rust-infected leaves on the lower surface of each leaf by stapler method of inoculation, then covered with polythene bags for 24 h to maintain humidity. The infected leaves stapled to healthy leaves for the period of 48 hrs and removed. Inoculated plants were maintained at 22–24 °C under natural light in the greenhouse. The reaction of pathogen on each host species was recorded as ‘Infection’ (+) or ‘No infection’ (-). The experiment was repeated thrice to confirm the inoculation procedure and also the presence of rust pustules. The alternate hosts were kept for ten days after inoculation to observe the presence or the absence of rust pustules.

Selected specimens (rust infected cotton leaves of Bt hybrids) were examined by FESEM (Zeiss SIGMA VP 03-04; Zeiss, Oberkochen, Germany) at South India Textile Research Association (SITRA), Coimbatore. To prepare samples for analysis, rust-infected leaf material was manually cut into small pieces (∼five × five mm) using a razor blade. The samples were then fixed in 2.5% glutaraldehyde in 0.02 M sodium phosphate buffer (pH 7.1) for 48 h, with 24 h at room temperature and another 24 h at 4 °C. This was followed by six washes, each lasting 30 min, in 0.02 M sodium phosphate buffer at 4 °C. The samples were subsequently dehydrated through a graded ethanol series (30, 40, 50, 60, 70, 85, 95, and 100% v/v), with each concentration applied twice for 30 min. For SEM analysis, the specimens underwent critical point drying in CO_2_ after fixation and dehydration, were mounted on carbon stubs, and coated with a 15 nm gold layer. The SEM analysis was conducted using an acceleration voltage of 20 kV (Alves et al., 2013).

The germination percentage of *P. gossypii* urediniospores was assessed across different incubation periods and temperatures. Rust spore suspension was incubated for different hours starting from 4 h to 72 h and then germination of spores was viewed under microscopy to calculate the percentage germination of spores. The incubation was carried out under dark conditions/controlled light regime, *e.g.*, 12 h photoperiod. Typically, dark conditions are preferred for rust spore germination studies, as light can inhibit or alter germination behavior in rust fungi. The use of a low concentration of Tween 20 (0.1%) was used, as it improves homogeneity by reducing spore clumping, thereby ensuring more consistent inoculum preparation also enhances reproducibility. Similarly, the spore suspension was stored at various temperatures *viz.,* 25 °C, 30 °C, 35 °C and 40 °C and observed for the germination percentage. Germinated spores were counted under ten different microscopic fields and average was recorded. In the incubation period experiment, temperature maintained was uniform (25 °C) for all the incubation periods. In the temperature experiment, incubation period was uniform (24 hrs) for different temperatures. Viability and survival of rust urediniospores were also examined under various storage conditions. Different storage conditions such as room 23 °C to 28 °C, under shade and under refrigerator conditions 4 °C were used to incubate rust spores for different durations *viz.,* 5, 10, 15, 25, 35 and 40 days. After each storage and days of incubation, percentage germination was observed. For all the studies on germination for different incubation periods, temperatures and viability, three replications were maintained for all the particular treatments and was repeated thrice to confirm the findings.

## Results

During the year 2022, the disease intensity/severity ranged from 12.52% to 55.11% in various Bt hybrids and varieties. The highest percent disease intensity was observed in hybrid RCH659-55.11% and in variety Suraj- 12.52%. By the year 2023, the disease intensity/severity ranged from 7.21% to 53.11% in various Bt hybrids and varieties. The highest percent disease intensity was observed in hybrid RCH659-53.11% and in variety Suraj- 7.21% (Table 1). Cotton leaves developed typical pustules of *P. gossypii*, confirming the viability and infectivity of urediniospores under favorable conditions. Among thirteen non-cotton host species tested, eight exhibited infection with pustules containing *P. gossypii* urediniospores (Table 2). Rust pustules were observed on *Arachis hypogaea, Abelmoschus esculentus, Vigna radiata, Vigna unguiculata, Phaseolus vulgaris, Glycine max, Helianthus annuus* and *Macrotyloma uniflorum*. However, *Sesamum indicum*, *Cajanus cajan*, *Lablab purpureus*, *Cyamopsis tetragonoloba*, *Cicer arietinum* showed no pustules during its incubation period.

**Table 1:** Percent disease index of rust disease in cotton.

**S. No**	**Hybrids & Varieties**	**2022** **Disease intensity % (140 DAS)**	**2023** **Disease intensity % (140 DAS)**
**Bt Hybrids**			
1.	RCH659 BG II	55.11 (47.93)	53.11 (46.78)
2.	Suraj Bt	42.71 (40.81)	40.71 (39.65)
3.	Rajat Bt	32.51 (34.76)	22.51 (28.32)
**Varieties**			
4.	Surabhi	45.14 (42.21)	25.14 (30.09)
5.	Anjali	42.54 (40.71)	22.54 (28.34)
6.	Sumangala	41.34 (40.01)	21.34 (27.51)
7.	MCU5VT	40.32 (39.42)	20.32 (26.79)
8.	LRA5166	38.14 (38.14)	28.14 (32.04)
9.	BB7	37.11 (37.53)	27.11 (31.38)
10.	Supriya	34.23 (35.81)	24.23 (29.49)
11.	Sunantha	29.11 (32.65)	19.11 (25.92)
12.	Subiksha	28.43 (32.22)	18.43 (25.42)
13.	CCH 15	27.33 (31.52)	24.33 (29.55)
14.	Suchitra	25.76 (30.50)	15.76 (23.39)
15.	Suraksha	15.34 (23.06)	15.34 (23.06)
16.	Suvin	34.21 (35.80)	14.21 (22.15)
17.	Nano	10.47 (18.88)	8.20 (16.64)
18.	CCH 2623	11.26 (19.61)	7.42 (15.81)
19.	Suraj	12.52 (20.72)	7.21 (15.58)
SD		**9.37**	**10.74**
S. Em. ±		**2.34**	**2.46**
CV (%)		**0.26**	**0.11**

**Notes.**

Figures in parentheses are transformed values.

DAS, Days after sowing.

**Table 2:** Host range studies of *P. gossypii*.

**S. No**	**Hosts**	**Infection**
1.	*Arachis hypogaea*, *Abelmoschus esculentus*, *Vigna radiata*, *Vigna unguiculata*, *Phaseolus vulgaris*, *Glycine max*, *Helianthus annuus and Macrotyloma uniflorum*	+
2.	*Sesamum indicum*, *Cajanus cajan*, *Lablab purpureus*, *Cyamopsis tetragonoloba*, *Cicer arietinum*	–

**Figure 1:**
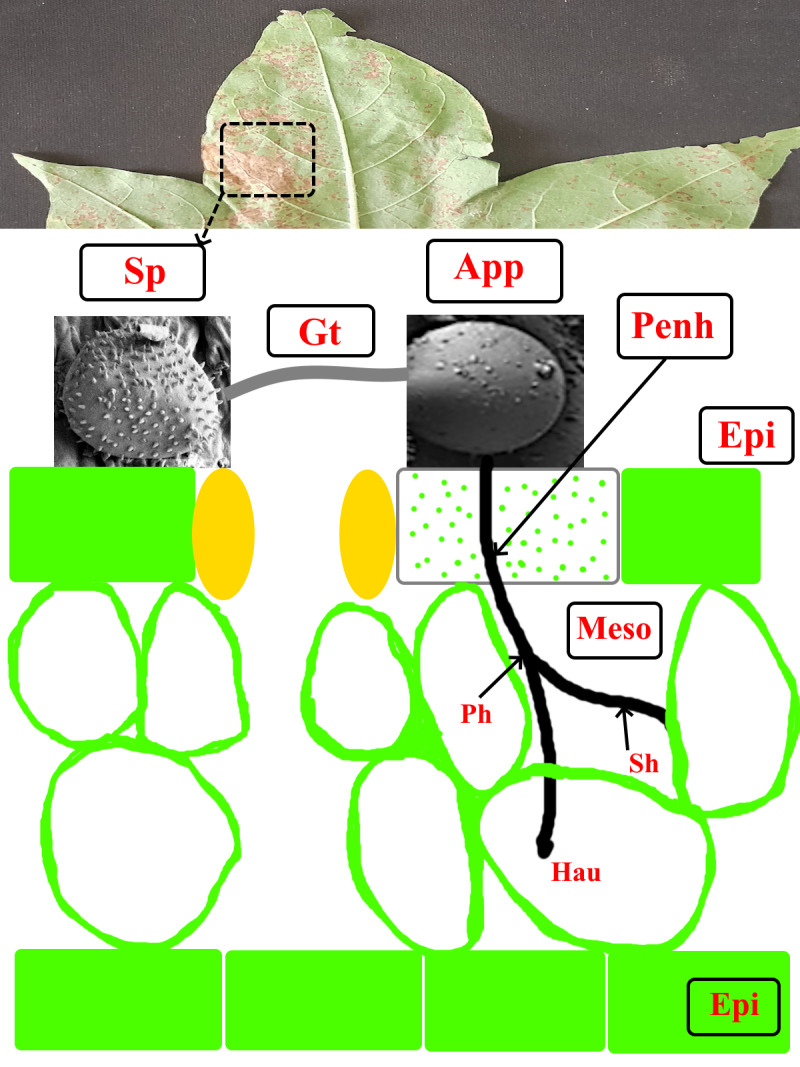
Initial developmental stages during interactions of *Phakopsora gossypii* with cotton. Sp, Germinating urediniospores; Gt, Germ tube; App, Appressorium; Penh, Penetration hypha; Epi, Epidermal cells; Ph, Primary hyphae; Sh, Secondary hyphae; Hau, First haustorium; Meso, Mesophyll.

**Figure 2:**
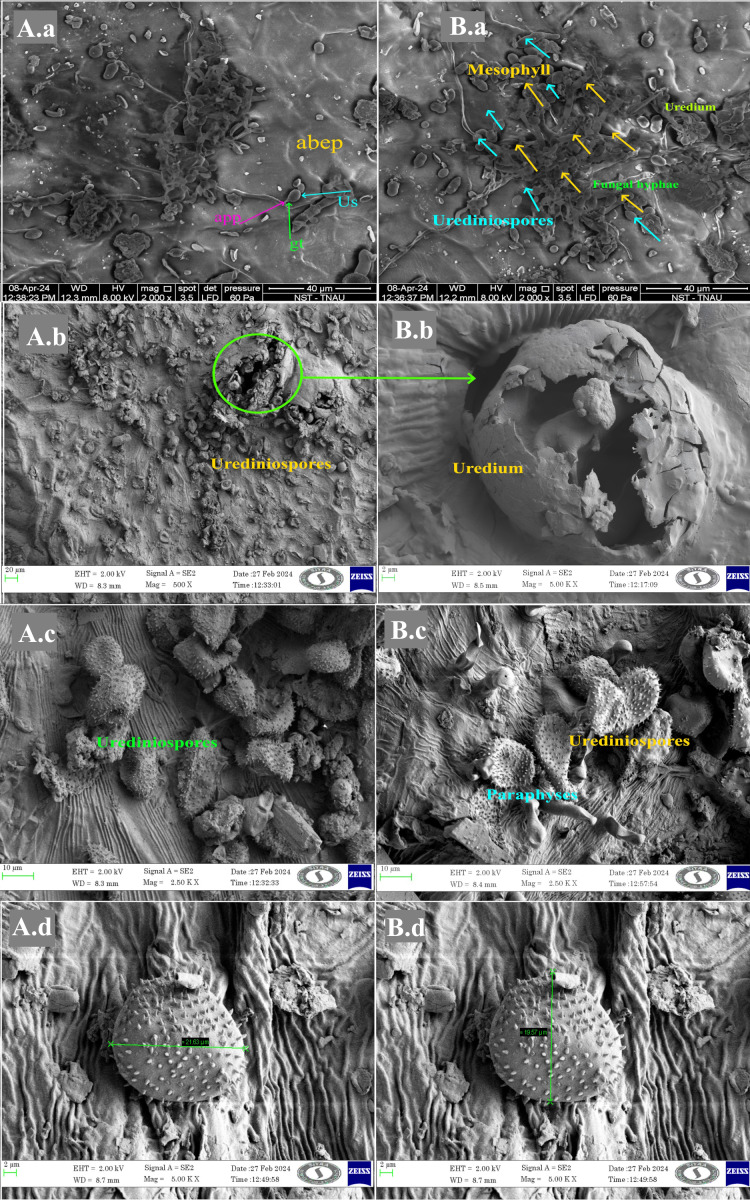
Infection process of *Phakopsora gossypii* as Scanning Electron Micrographs (SEM). (A.a & B.a) Germination of *Phakopsora gossypii* urediniospore on the abaxial surface of cotton leaf at 42 h after inoculation with germ tube and appressorium formation. Urediniospore (us), germ tube (gt), abaxial epidermis (abep), and appressorium (app). Scale bars = 40 µm. (A.b) Urediniospores after 20 DAI with *P. gossypii*, (B.b) View of erumpent uredium on the abaxial leaf surface of cotton. Scale bars = 20 µm and 2 µm (A.c) Top view of urediniospores with echinulate surface; (B.c) Urediniospores along with paraphyses at 35 DAI with *P. gossypii*. Scale bars = 10 µm Size of Urediniospores (A.d & B.d) Width-21.63 µm (B4) Length-19.57 µm. Scale bars = 2 µm.

During early interactions with cotton, *P. gossypii* produces asexual urediniospores on short stalks within a uredium 5 to 8 days after inoculation. Urediniospores are released through an ostiole and dispersed by wind. Under suitable conditions, germinating urediniospores (*Sp*) produce a single germ tube (*Gt*), which forms a globose appressorium (*App*). This develops into a penetration hypha (*Penh*) that crosses epidermal cells (*Epi*). In the mesophyll’s intercellular space, a primary hypha (*Ph*) emerges, separated by a septum, and branches into secondary hyphae (*Sh*). The pathogen then invades a mesophyll cell (*Meso*), forming the first haustorium (*Hau*) to establish infection (Fig. 1). It is stated that this figure is the hypothesised behaviour of *P. gossypii* based on knowledge for *P. pachyrhizi*. The infection process of *Phakopsora gossypii* as SEM figures has been depicted in Fig. 2. In the case of *Phakopsora gossypii*, the urediniospores germinated which produces a germ tube and an appressorium at 42 h after inoculation (HAI) that potentially allows the fungus to breach the leaf cuticle directly (A.a & B.a). At 20 days after inoculation (DAI), on the abaxial leaf surface, closed uredia containing urediniospores were observed (A.b & B.b). By 25 DAI, the uredia started to open and were fully mature by 35 DAI, revealing numerous urediniospores which is of subglobose and elliptical in shape. Urediniospores along with sterile hair-like structure, Paraphyses in an open uredium was observed (A.c & B.c). Size of rust urediniospore reveals with width of 21.63 µm and length of 19.57 µm (A.d & B.d).

**Figure 3:**
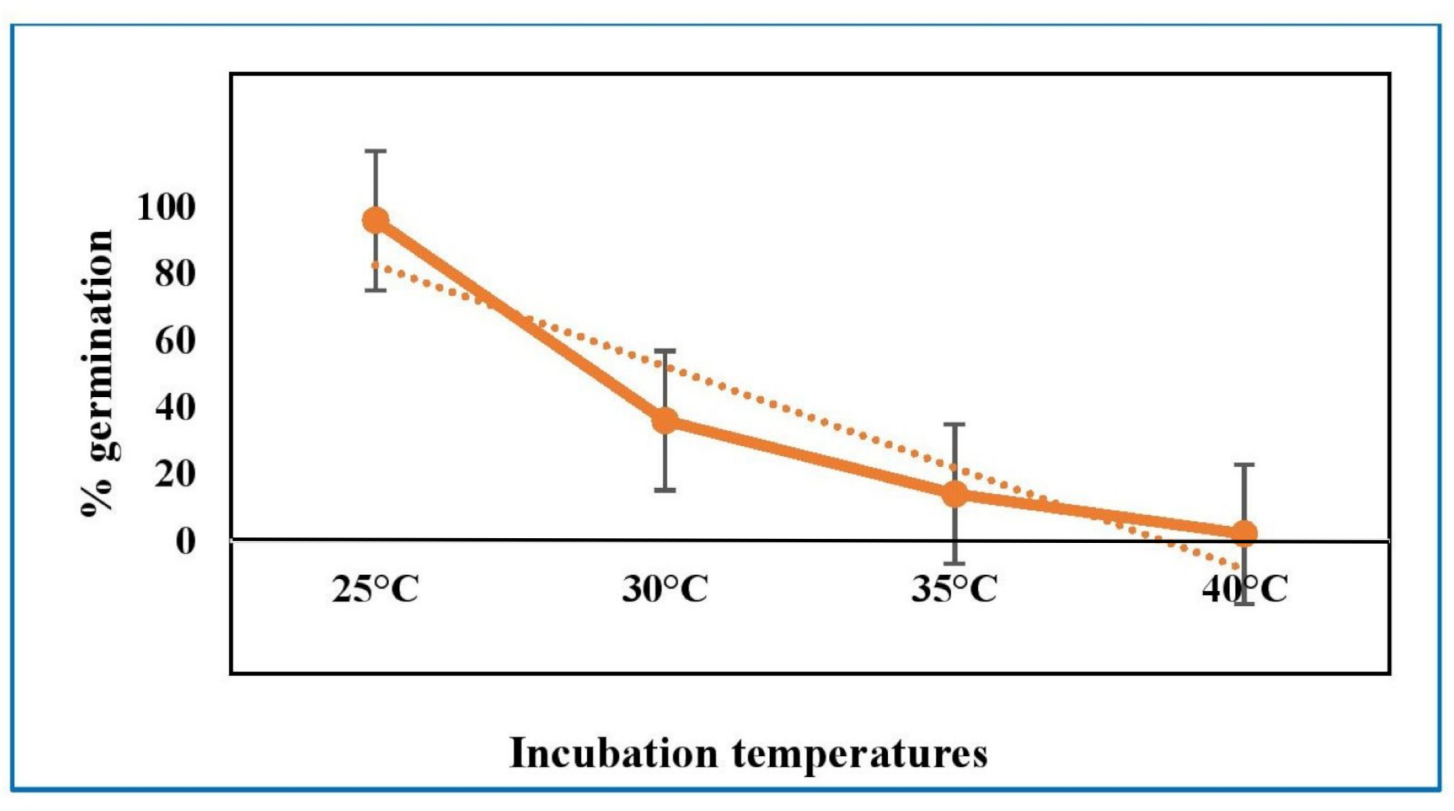
Percentage germination of *P. gossypii* urediniospores at different incubation temperature.

**Figure 4:**
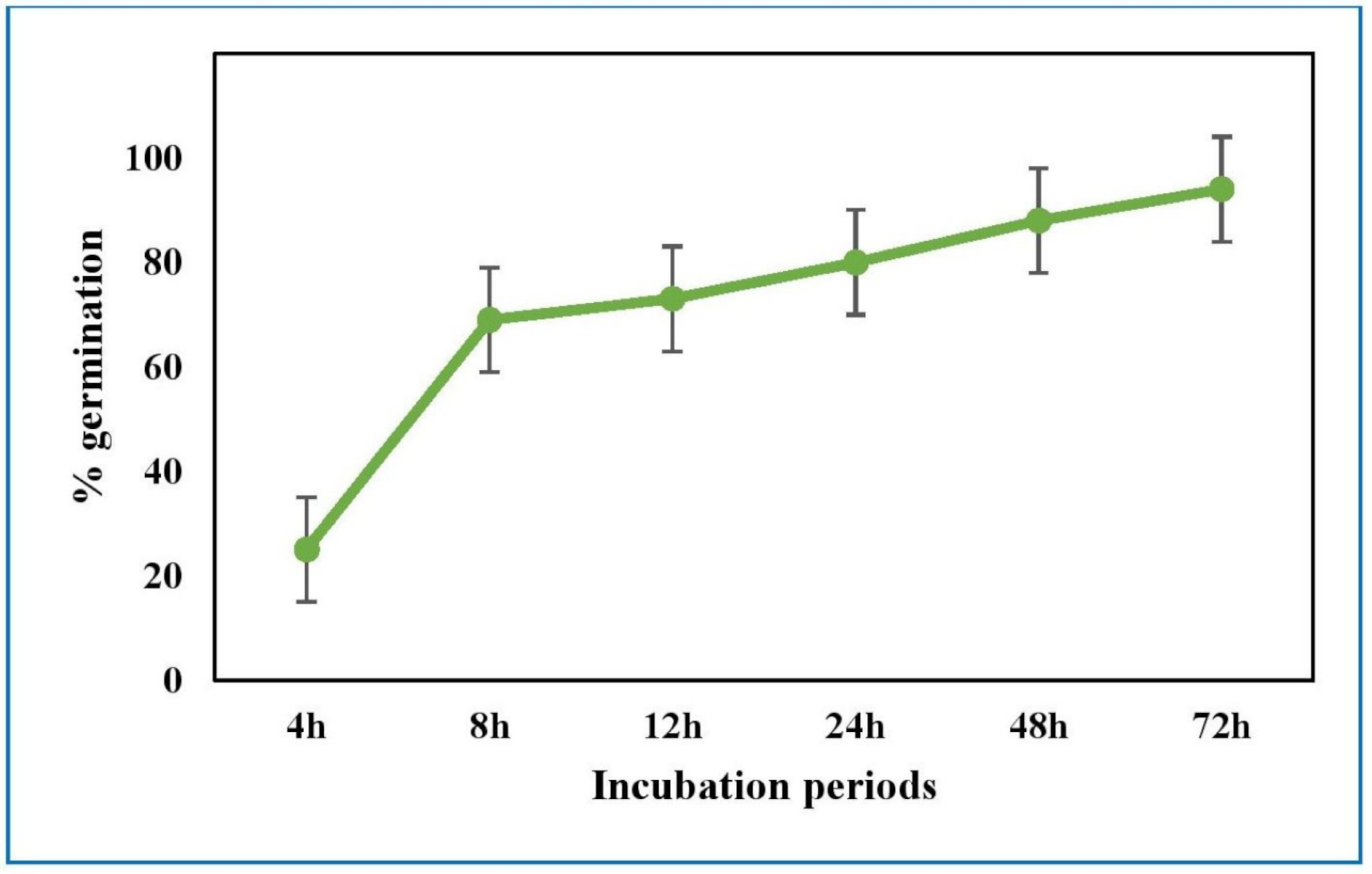
Percentage germination of *P. gossypii* urediniospores at different incubation periods.

Maximum germination (96.40%) was recorded at 24 h when stored at 25 °C, while higher temperatures reduced germination to 36.12% at 30 °C, 13.54% at 35 °C, and only 2.15% at 40 °C suggesting temperatures above 25 °C are unfavorable for urediniospore germination (Table 3a, Fig. 3). Spore germination is influenced by both temperatures and the duration of the incubation periods. Urediniospore germination began early (25.30% at 4 h) and increased over time, reaching 69.40% at 8 h (Table 3b). The highest germination (94.35%) occurred at 72 h (Fig. 4).

**Table 3:** Percentage germination of *P. gossypii* urediniospores.

**(a) Different incubation temperatures**					
**S. No**	**Details**	**25 °C**	**30 °C**	**35 °C**	**40 °C**
1.	Spore suspension (%)	96.40 (70.06)	36.12 (36.94)	13.54 (21.59)	2.15 (8.43)

**(b) Different incubation periods**							
**S. No**	**Details**	**4 h**	**8 h**	**12 h**	**24 h**	**48 h**	**72 h**
1.	Spore suspension (%)	25.30 (30.20)	69.40 (56.42)	73.20 (58.82)	80.30 (63.65)	88.25 (69.95)	94.35 (76.25)

**Notes.**

Figures in parentheses are transformed values.

In the temperature experiment incubation period was uniform (24 hrs) for different temperatures.

In the incubation period experiment temperature maintained was uniform (25 °C) for all the incubation periods.

**Figure 5:**
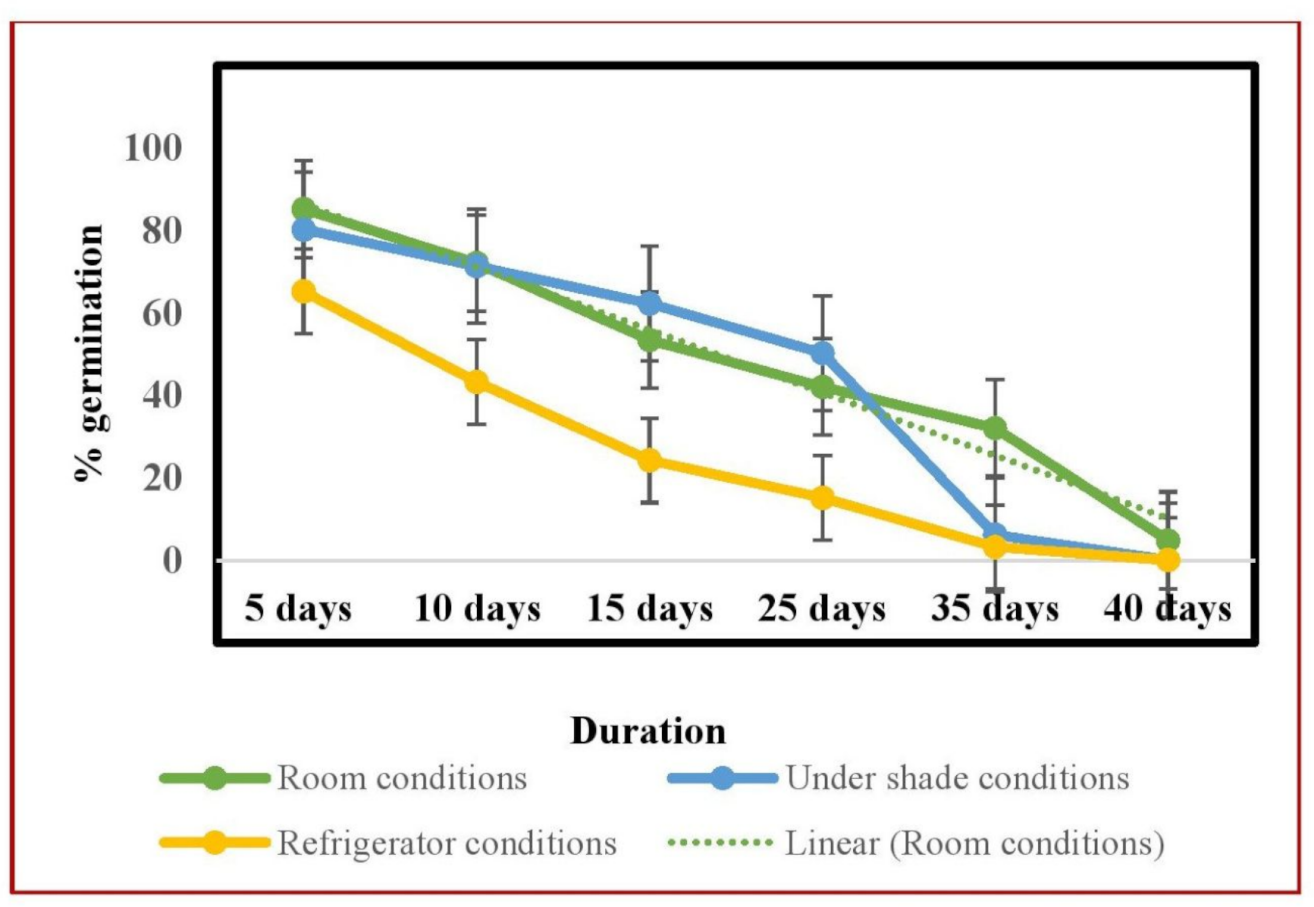
Viability and survival of *P. gossypii* urediniospores under different storage conditions.

Three different conditions for storage of spores taken as room, under shade and refrigerator conditions (Fig. 5). Urediniospore viability declined over time under all storage conditions. At room temperature, viability decreased from 85.20% to 4.80% over 40 days. Under shade, it lasted up to 35 days, dropping from 80.25% to 6.2%. In refrigeration, viability also persisted for 35 days but declined from 65.15% to 3.2%. No germination occurred after 45 days in any condition, with the lowest viability (3.2%) recorded in refrigeration after 35 days (Table 4).

## Discussion

The rust pustules formed as uredia on the leaves, appearing as small (1–3 mm) pinkish-brown spots surrounded by a purple halo. Similar symptoms were also observed on petioles, stems, and bolls, where uredia appeared. The presence of rust pustules led to a reduced photosynthetic area in leaves, ultimately resulting in severe defoliation in the field and a subsequent decrease in cotton yield. A study was conducted to evaluate the survival of the pathogen through urediniospores on various hosts in the absence of the primary host. Given the vital importance of cotton rust and the need for wider understanding of its underlying factors to develop more effective control strategies, the research focused on examining the infection process of *Phakopsora gossypii* in cotton leaves using Scanning Electron Microscopy (SEM) and also explored the survival and viability of urediniospores. It is stated that figure initial developmental stages during interactions of *P. gossypii* with cotton is the hypothesised behaviour of this pathogen based on knowledge gained from *P. pachyrhizi* (Goellner et al., 2010). In the field, *Phakopsora pachyrhizi* infects a wide range of at least 31 species across 17 genera of leguminous plants. Additionally, under laboratory conditions, it has been shown to infect 60 more species from other genera (Goellner et al., 2010). Hegde (2001) reported that *P. pachyrhizi* could infect crops such as *Saccharum officinarum, Gossypium hirsutum, Arachis hypogaea, Phaseolus vulgaris, Cajanus cajan, Vigna radiata, Vigna unguiculata* and *Macrotyloma uniflorum*. Furthermore, he found that *Phaseolus vulgaris* and *Psophocarpus tetragonolobus* were the most susceptible, exhibiting the highest average number of pustules per leaf, whereas, *Macrotyloma uniflorum*, *Saccharum officinarum, Cassia*, *Cyperus rotundus* and *Stylosanthes* had the lowest. However, in the present study, cotton and soybean were identified as highly susceptible to the *P. gossypii*. Similar observations on the host range for cotton rust was reported by Pindikur et al. (2012). The hosts susceptible to *P. gossypii* provide a means for the pathogen to survive even when its primary host is absent and provide source of inoculum for the growing season. Therefore, eliminating these secondary hosts near cotton fields could prevent the onset and dissemination of rust on cotton plants.

**Table 4:** Viability and survival of *P. gossypii* urediniospores under different storage conditions.

**S. No**	**Storage conditions**	**5 days**	**10 days**	**15 days**	**25 days**	**35 days**	**40 days**
1.	Room	85.20% (67.37)	72.10% (58.12)	53.42% (49.96)	42.10% (40.45)	32.10% (34.51)	4.80% (12.66)
2.	Under shade	80.25% (63.61)	71.35% (57.64)	62.34% (52.14)	50.24% (45.14)	6.2% (14.42)	—
3.	Refrigerator	65.15% (53.82)	43.27% (41.13)	24.20% (29.47)	15.20% (22.95)	3.2% (10.30)	—

**Notes.**

Figures in parentheses are transformed values.

Mayee (1987) experimented with altering environmental conditions to induce telial formation in cotton but was unsuccessful. According to his research, urediniospores were identified as the primary, if not sole, method of rust propagation and dissemination in India. Srinivasan (1994) noted that only the uredial stage of cotton rust is prevalent in India. Although initial phases of telial formation have been observed, they have not matured, whereas in *P. pachyrhizi* germination of teliospore and formation of basidiospore can be successfully induced under laboratory conditions (Saksirirat & Hoppe, 1991). However, the infection process of basidial spores remains unknown, as does whether *P. pachyrhizi* is autoecious or heteroecious. Additionally, spermatia and aeciospores typically associated with host alternation in heteroecious rusts, have not been identified in this fungus (Bromfield & Hartwig, 1980). From our study, it is inferred that only uredial stage of cotton rust has been observed.

Guerra et al. (2013) found that cotton rust takes between 20 to 22 days after infection (DAI) to end its latent period on cotton plants of the cv. BRS Buriti. Medice et al. (2007) observed that the uredia of *P. pachyrhizi* on soybean leaves fully opened at 36 DAI. Usually, the smaller, closed uredia of pathogen on soybean leaves are associated to reduced symptoms of rust due to their lower production of urediniospores (Cruz et al., 2012; Medice et al., 2007; Vittal et al., 2014). Based on SEM observations, Cruz et al. (2012) reported that the fungal hyphae densely colonized the parenchyma cells adjacent to the uredial spores of *P. pachyrhizi* in soybean leaves.

Vittal et al. (2014) reported that for a compatible soybean-*P. pachyrhizi* interaction, extensive fungal hyphae colonization of the mesophyll is essential. Edwards & Bonde (2011) used transmission electron microscopy and observed the development of hyphae and haustorial mother cells of the soybean rust pathogen in the soybean mesophyll between 14 and 21 DAI. In *Phakopsora gossypii*, urediniospores germinated by 42 h after inoculation, forming germ tubes and appressoria that likely facilitated direct penetration of the leaf cuticle. By 20 days after inoculation (DAI), closed uredia containing urediniospores were visible on the abaxial leaf surface. Uredia began to open at 25 DAI and reached full maturity by 35 DAI, releasing abundant urediniospores (Araujo et al., 2015). In this study, the colonization pattern of *P. gossypii* in cotton resembled that of *P. pachyrhizi* in soybean. This study’s findings suggest that effective cotton rust management, including fungicide application, should begin within 20 days of infection. From our study, it is revealed that *Phakopsora gossypii*, the urediniospores germinated which produces a germ tube and an appressorium at 42 h after inoculation and found to have matured uredia with spores at 35 DAI. Delaying beyond this timeframe allows the uredia to open and release initial urediniospores, which disseminate across cotton leaves as the spores are air-borne in nature.

The present findings observed were in similar to results obtained from the studies carried out by Pindikur et al. (2012) as maximum uredospore germination (95.62%) occurred at 25 °C after 24 h. Germination decreased at 30 °C (35.10%) and was markedly reduced at 35 °C (12.89%) and 40 °C (1.13%), indicating that temperatures above 25 °C are unfavorable for germination. Temperature, humidity and incubation period are the factors which influences the germination of spores. Germination of urediniospore at different incubation periods in the laboratory shows that the germination of urediniospores started initially (25.30% at 4 h) and increases over time with 69.40% after 8 h of incubation. Maximum germination (94.35%) was observed at 72 h of incubation. The highest germination rate of urediniospores (96.40%) was observed after 24 h when stored at 25 °C. However, when subjected to a higher temperature of 30 °C, the germination rate decreased to 36.12% within the same time frame. Moreover, at even higher temperatures of 35 °C and 40 °C, the percentage of germination decreased further to 13.54% and 2.15% respectively. These findings suggest that temperatures above 25 °C may not be favourable for urediniospore germination.

Patil et al. (1997) revealed that *P. pachyrhizi* urediniospores when exposed under natural open conditions with temperatures ranging from 28 °C to 30 °C, the urediniospores lost their viability within 25 days. Similarly, when kept at a constant temperature of 28 °C under incubator conditions, their viability decreased within 30 days. The urediniospores remained viable in a wooden cage outside the laboratory (25−28 °C) for 40 days, 45 days under laboratory conditions (20−25 °C), and 55 days in the shade of a tree (15−20 °C). Hundekar (1999) explained that the urediniospores exhibited a short lifespan of 12 to 15 days when present in infected host debris, regardless of the environmental conditions. Hegde (2001) reported that *P. pachyrhizi* urediniospores when exposed to field conditions with temperatures ranging from 28 °C to 30 °C, as well as glasshouse conditions between 25 °C and 28 °C, their viability declined within 15 days. However, under shade conditions with temperatures between 15 °C and 20 °C, the urediniospores remained viable for 35 days. Furthermore, they retained viability for an extended period of 40 days when incubated at room temperature of 20 °C to 25 °C. Under simulated Southern Louisiana winter conditions (12 °C with 14-hour days and 1 °C with 10-hour nights at 75% relative humidity), the viability of soybean rust urediniospores reduced rapidly from 72% to 40% within one day. Over time, it slowly decreased to 17% after seven days and further dropped to 11% after 60 days (Park et al., 2008).

Temperature and relative humidity (RH) played a crucial role in the survival of urediniospores, as reflected in germination rates. Urediniospores collected from diseased soybean leaves and stored at room temperature (23–24 °C with 55–60% RH) remained viable for upto 18 days. Moreover, freshly collected urediniospores that were desiccated for 12 h before being placed in vials and kept at room temperature retained viability for up to 30 days. (Twizeyimana & Hartman, 2010). From the current study, it was observed that viability of urediniospores of *P. gossypii* decreased over time under all storage conditions. At room temperature, viability lasted up to 40 days, declining from 85.20% to 4.80%. Under shade, viability lasted up to 35 days (80.25% to 6.2%). In the refrigerator, viability lasted up to 35 days (65.15% to 3.2%). No germination occurred after 45 days under any storage condition. The lowest viability (3.2%) was observed in the refrigerator after 35 days of storage.

Based on the ongoing discussion, it can be inferred that the highest germination of *P. gossypii* urediniospores causing rust in cotton occurred after 72 h of incubation, within a temperature range of 20–25 °C. These conditions are optimal for infection and disease progression. If temperature deviate from this range, disease development either slows down or remains dormant until suitable conditions to revive. Understanding these dynamics, will aid in developing effective management strategies, especially in the context of climate change. The present study also shows the long period for which urediniospores can remain viable on a range of different storage and under a wide range of temperature conditions and highlights the importance of its survival in the cotton.

## Conclusion

In conclusion, the results of the present study provide novel information regarding the infection process of *P. gossypii* in cotton leaves, which might be useful in the development of new and more effective management strategies to control cotton rust. The early infection processes, including appressorium-mediated penetration and colonization, with deeper ultrastructural studies has been addressed. Despite progress in understanding cotton rust disease, several critical knowledge gaps remain to be addressed in future. There is limited insight into the molecular mechanisms governing host–pathogen interactions, particularly effector biology and resistance gene identification. Comprehensive studies on pathogen genetic diversity, race structure, and regional variability are lacking, constraining the development of durable resistance. Additionally, the mechanisms underlying host resistance and their integration into genomics-assisted breeding programs remain insufficiently explored. Predictive epidemiological models incorporating environmental and climate variables are underdeveloped, limiting effective disease forecasting. Enhanced epidemiological investigations are essential for the development of a weather-based forecasting model, which can be integrated into the Appropriate Integrated Management System. Addressing these gaps for future is essential for developing sustainable and scientifically sound management strategies for cotton rust disease.

## Supplemental Information

10.7717/peerj.21412/supp-1 Supplemental Information 1 Raw data.

## Additional Information and Declarations

### Data Availability

The following information was supplied regarding data availability: The raw data is available in the Supplemental File.
